# Projections onto Convex Sets Super-Resolution Reconstruction Based on Point Spread Function Estimation of Low-Resolution Remote Sensing Images

**DOI:** 10.3390/s17020362

**Published:** 2017-02-13

**Authors:** Chong Fan, Chaoyun Wu, Grand Li, Jun Ma

**Affiliations:** 1 School of Geosciences and Info-Physics, Central South University, Changsha 410083, China; fanchong@csu.edu.cn; 2 State Key Laboratory of Information Engineering in Surveying, Mapping and Remote Sensing, Wuhan University, Wuhan 430079, China; 3 SiChuan Remote Sensing Geomatics Institute, NO. 2, Jianshe Road, Longquanyi District, Chengdu 610100, China; grandl@126.com

**Keywords:** super resolution, point spread function (PSF), projections onto convex sets (POCS), remote sensing

## Abstract

To solve the problem on inaccuracy when estimating the point spread function (PSF) of the ideal original image in traditional projection onto convex set (POCS) super-resolution (SR) reconstruction, this paper presents an improved POCS SR algorithm based on PSF estimation of low-resolution (LR) remote sensing images. The proposed algorithm can improve the spatial resolution of the image and benefit agricultural crop visual interpolation. The PSF of the high-resolution (HR) image is unknown in reality. Therefore, analysis of the relationship between the PSF of the HR image and the PSF of the LR image is important to estimate the PSF of the HR image by using multiple LR images. In this study, the linear relationship between the PSFs of the HR and LR images can be proven. In addition, the novel slant knife-edge method is employed, which can improve the accuracy of the PSF estimation of LR images. Finally, the proposed method is applied to reconstruct airborne digital sensor 40 (ADS40) three-line array images and the overlapped areas of two adjacent GF-2 images by embedding the estimated PSF of the HR image to the original POCS SR algorithm. Experimental results show that the proposed method yields higher quality of reconstructed images than that produced by the blind SR method and the bicubic interpolation method.

## 1. Introduction

Image resolution refers to the number of pixels contained in an image per unit area. This parameter is an important factor used to evaluate the quality of remote sensing images. The limitations of imaging systems and the external circumstances in obtaining images, including inherent sensor sampling frequency, defocusing, and atmospheric disturbances [1], result in low-quality images, which are blurred, misshapen, and exhibiting random noise. To solve these problems, two methods were proposed. First, the resolution can be enhanced by increasing the chip size. However, this approach is costly and cannot significantly improve the image resolution. Second, super-resolution (SR) reconstruction can be implemented using various algorithms. In the present study, time is used to compensate for space. Certain constraints or algorithms are employed to build a high-resolution (HR) image with higher number of pixels, more details, and better image quality than those of the observed multiple low-resolution (LR) images [2]. The proposed approach demonstrates the advantage of obtaining huge information at minimum economic cost; hence, this approach has been widely used in many applications such as military monitoring and medical diagnosis. Moreover, SR algorithms provide a wide range of applications in agriculture. Kasturiwala reported that the SR reconstruction method can estimate some missing high-frequency details from an infected leaf image [3]. This method is most useful to agricultural experts in helping farmers detect exact leaf diseases and provide accurate remedial actions. In [4], researchers introduced a SR mapping method to produce a fine-spatial-resolution land cover map from coarse-spatial resolution remotely sensed imagery. The result of the SR reconstruction will be affected by the accuracy of the point spread function (PSF) estimation of images. Therefore, the research in [5] investigates the adequacy of a remote sensing instrument spatial resolution in monitoring crop growth in agricultural landscapes with different spatial patterns. Furthermore, the studies on SR reconstruction and the relationship between PSF in the HR and the LR images provide significant findings.

The concept of SR reconstruction was presented in 1965 when the Harris–Goodman spectrum extrapolation method was proposed by Harris [6] and Goodman [7], and this concept has become a popular research topic in image processing. Reconstruction methods classified according to the numbers of LR images can be divided into two categories: reconstruction based on a single-frame image and reconstruction based on a sequence of images. Many of these algorithms utilize a single-frame LR input image reconstructed into HR image by modeling; these algorithms also use the matching mechanisms or some prior information about the image [8]. The HR image is estimated from a sequence of LR aliased images of the same object or scene [9,10]. These algorithms generally reconstruct an image with enhanced resolution, which exhibits tighter pixel density and better image detail, by using the aliased information of multiple LR images.

The current study focuses on SR reconstruction by using the information obtained from multiple LR images. The popular methods include frequency domain and spatial domain algorithms. The first method is based on the shift property of the Fourier transform. The image is converted into the frequency domain to eliminate spectrum aliasing, obtain much of the missing high-frequency information, and improve the spatial resolution of the image. In particular, the popular methods mainly include spectral de-aliasing reconstruction algorithm, recursive least square method, and generalized sampling scheme. Frequency-domain algorithms are superior because they follow a simple theory and can be applied completely in parallel. However, these algorithms present the limitation of ignoring the prior knowledge in the spatial domain. The SR reconstruction approach uses complex observation models, which consider some spatial factors affecting the quality of the image, such as optical blur and motion blur, to reconstruct the image. Irani and Peleg [11,12] proposed the iterative back projection (IBP) algorithm. This approach can estimate the initial value of the HR image through some interpolation algorithms on a sequence of observed LR images. A set of simulated LR image arrays can be obtained comprehensively from the HR image by using the blurring model. Subsequently, the algorithm compares the observed LR images and the estimated LR images to achieve the correction value by taking a number of iterations to obtain the final HR image. However, the accuracy of the approach is low because the solution is not unique, and the prior information is difficult to apply. Stark and Oskoui [13] proposed the projection onto convex sets (POCS) method in 1987. In this method, a convex model considers a set of constraints that limit the SR feasible solutions (such as smoothness, energy boundedness, and consistency of data observations), and the intersection of sets is the final solution. POCS has become a significant method to solve the SR reconstruction problem. In the following years, the maximum likelihood (ML) algorithm [14] has been explored. Statistics showed that the method can provide an HR image through the expectation maximum algorithm. The maximum a posteriori probability (MAP) [15,16] is another SR approach with some mixed SR reconstruction methods (MAP/POCS algorithm) [17].

POCS algorithm considers a variety of degraded factors, including blur and movement; hence, this algorithm is important in solving the problem in SR reconstruction. Recently, Ogawa [18] proposed the POCS algorithm based on principal component analysis (PCA). Xi [19] improved the initial image estimation by using the wavelet bicubic interpolation for POCS reconstruction algorithm; the experimental results are evident. Liang [20] presented a POCS algorithm based on text features; in this method, text features are added as constraints to preserve the edge details and smoothen the noise in the text images. Meanwhile, the blind SR method is proposed by Sroubek and Flusser [21] can incorporates blur estimation into SR by performing an advanced deconvolution task. The model is built by the sharpness of edge regions and the smoothness of smooth regions in the total variance of the image, as well as the prior information of the image ambiguity function. Subsequently, the cross-iteration method is used to solve the model, and then the PSF and HR image are obtained. Thus, the HR image can be reconstructed even if the degradation model and the model’s parameters of the camera sensors are unknown [22,23]. Given that the methods of estimating PSF are different, two algorithms are used for reconstruction to compare the results.

In this work, an improved POCS SR algorithm based on PSF estimation of LR remote sensing images is proposed. Exploration of the relationship between the PSF of the HR image and the PSF of multiple LR images is an essential part of this algorithm. In this study, the formula is deduced and approved by the experiments conducted. The conclusions are provided in the succeeding section. Moreover, the estimated PSF of the HR is embedded to the original POCS SR algorithm, and the reconstruction results of the three different SR methods (proposed method, blind SR method, and bicubic interpolation method), in the simulated experiment and the real experiment, are compared.

## 2. Materials and Methods

### 2.1. Observation Model

**Figure 1:**
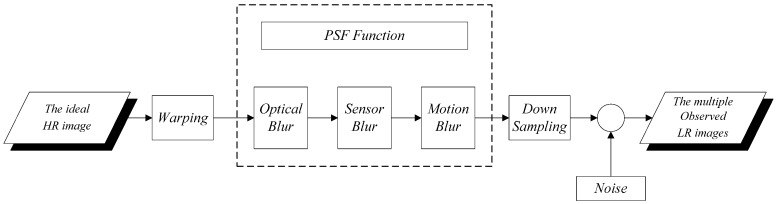
Observation model.

Generally, SR image reconstruction techniques present an inverse problem of recovering the HR image by degrading the LR images. The HR image is obtained under certain conditions, such as satisfying the theory of Nyquist sampling, and is affected by some of the inherent resolution limitations of sensors in the acquisition process [24], including warping, blurring, subsampling operators, and additive noise. Therefore, an observation model (1) can be formulated, which relates to the ideal HR image f to the corresponding i-th observed LR images $g_{i}$. The model can overcome the inherent resolution limitation of the LR imaging systems. The LR images display different subpixel shifts from each other because the spatial resolution is very low to capture all the details of the original scene. Finally, a goal image with denser pixel values and rich image information, called HR image, will be achieved:

(1) $$g_{i}=D_{i}H_{i}B_{i}f+n$$

where f is the ideal undegraded image required to be calculated, $g_{i}$ is the observed LR image, and $H_{i}$ represents the blur matrix, including relative camera-scene motion blur, sensor blur, atmospheric turbulence, and optical blur. Generally, the blur matrix is modeled as convolution with an unknown PSF to estimate blurs [25]. $B_{i}$ is the warp matrix (e.g., rotation, translation scaling, and so on). The relative movement parameters can be estimated using the subpixel shifts of the multiple LR images. $D_{i}$ represents a subsampling matrix, and n is the lexicographically ordered noise vector. The observation model is illustrated in Figure 1.

Image degradation occurs when the acquired image is corrupted by many factors. The image egradation process can be viewed as a linear invariant system, in which the noises can be ignored. The degradation model is described by Equation (2):

(2) φ= H* γ+ η ≈ H* γ

The model is composed of four main attributes: the original image without degradation γ, the degraded image φ, a PSF H, and some noises η. * is the convolution operating symbol. To restore the quality of the image, H can be estimated using some PSF estimation methods, such as the knife-edge method. When the original image is processed by downsampling, the PSF h of the downsampled image is obtained. However, h does not apply to the model (2); hence, the relationship between H and h is derived in Section 2.3. The derived formula will be applied to the SR reconstruction model.

### 2.2. Principle of POCS SR Algorithm

In this work, the POCS SR reconstruction algorithm is used to obtain high-quality remote sensing data, which can meet the requirements of agricultural data sources. The algorithm, which is simple and effective, is a collection theory of the image reconstruction method. Given the flexible space-domain observation model and the powerful prior knowledge embedding capability, the owned feasible region of the reconstructed image consists of an intersection consistency projective convexity set and a convex constraint set. The POCS algorithm [26] is an iterative operation; the operator of the corresponding convex constraint set projects the points in the solution space to the nearest point on the surface of the convex set. After a finite number of iterations, a solution to the intersection set that converges to the convex constraint set is finally found. The POCS SR algorithm is detailed as follows:

*Step 1*: Estimate the image $f_{0}$ by using the linear interpolation method for LR images.
*Step 2*: Compute the motion compensation of the pixel of each LR image. The correspondence between the LR image and the HR image is given by Equation (3):
  (3) $$g\left(m_{1},m_{2},l\right)=\;\underset{n_{1},n_{2}}{\sum }f\left(n_{1},n_{2}\right)\;h\left(n_{1},n_{2};m_{1}^{\prime },m_{2}^{\prime },l\right)+n\left(m_{1},m_{2},l\right)$$
  where ($m_{1},m_{2})$ is the point in the LR image, and $\left(n_{1},n_{2}\right)$ is the corresponding point in the HR image.
  1. Obtain the position of the pixel on the LR image of each frame $g\left(m_{1},m_{2},l\right)$ and on the HR image $f\left(n_{1},n_{2}\right)$.
  2. Calculate the parameter, $h\left(n_{1},n_{2};m_{1}^{\prime },m_{2}^{\prime },l\right)$, which represents the range and the value of PSF according to the position of the pixel.
  3. Simulate the sampling process to obtain the simulated LR image. The observed LR image $g\left(m_{1},m_{2},l\right)$ can be constrained by a convex set $C_{n_{1},n_{1},k}$, as follows:
    (4) $$C_{n_{1},n_{1},k}=\left\{f\left(m_{1},m_{2},l\right):\left|r^{\left(f\right)}\left(n_{1},n_{2},k\right)\leq \delta_{0}\left(n_{1},n_{2},k\right)\right|\right\}\;0\leq n_{1},n_{2}\leq N-1,k=1,\cdots ,L$$
    The projection $P\left(n_{1},n_{2},k\right)\left[x\left(m_{1},m_{2},l\right)\right]$ at any point $x\left(m_{1},m_{2},l\right)$ on $C\left(n_{1},n_{2},k\right)$ is defined in Equation (5) as follows:
    (5) $$P\left(n_{1},n_{2},k\right)\left[x\left(m_{1},m_{2},l\right)\right]=\{\begin{array}{c} x\left(m_{1},m_{2},l\right)+\frac{r^{\left(x\right)}(n_{1},n_{2},k)-\delta_{0}(n_{1},n_{2},k)}{\sum_{o_{1}}\sum_{o_{2}}h^{2}(n_{1},n_{2};o_{1},o_{2},k)}h(n_{1},n_{2};m_{1},m_{2},l)\\ r^{\left(x\right)}(n_{1},n_{2},k)>\delta_{0}(n_{1},n_{2},k)\\ x(m_{1},m_{2},l)\\ -\delta_{0}(n_{1},n_{2},k)<r^{\left(x\right)}(n_{1},n_{2},k)<\delta_{0}(n_{1},n_{2},k)\\ x\left(m_{1},m_{2},l\right)+\frac{r^{\left(x\right)}(n_{1},n_{2},k)-\delta_{0}(n_{1},n_{2},k)}{\sum_{o_{1}}\sum_{o_{2}}h^{2}(n_{1},n_{2};o_{1},o_{2},k)}h(n_{1},n_{2};m_{1},m_{2},l)\\ r^{\left(x\right)}(n_{1},n_{2},k)<-\delta_{0}(n_{1},n_{2},k) \end{array}$$
  4. Calculate the residuals $r^{f}\left(n_{1},n_{2},k\right)\;$ between the real image and the simulated image. The formula can be described by (6).
    (6) $$r^{\left(f\right)}\left(n_{1},n_{2},k\right)=g\left(n_{1},n_{2},k\right)-\sum f\left(m_{1},m_{2},l\right)\cdot \;h\left(n_{1},n_{2};m_{1},m_{2},l\right)$$
    where $h\left(n_{1},n_{2};m_{1},m_{2},l\right)\;$is the impulse response coefficient, $\delta_{0}\;$is the confidence level on the observed result. In this paper, $\delta_{0}=c\;\delta_{v}$, where the point $\;\delta_{v}\;$is the standard deviation of the noise, and c≥ 0 is determined by an appropriate statistical confidence range. These settings define HR images that are consistent with the observed LR image frames within a certain confidence range proportional to the observed noise variation.
  5. Correct the pixel value of the HR image according to the residuals.
*Step 3*: Repeat from Step 2 until convergence

Given a projection operator, the estimated value $\hat{f}\left(m_{1},m_{2},l\right)$ of the HR image $f\left(m_{1},m_{2},l\right)$ can be obtained from all the LR images $g\left(n_{1},n_{2},k\right)$ through many iterations, such as Equation (7):

(7) $${\hat{f}}^{\left(i+1\right)}\left(m_{1},m_{2},l\right)=T_{\lambda }\tilde{T}\left[{\hat{f}}^{\left(i\right)}\left(m_{1},m_{2},l\right)\right]i=0,1,\cdots$$

where $\tilde{T}$ is the combination of all the relaxation projection operators associated with $C\left(n_{1},n_{2},k\right).$ The initial estimate, $f^{0}\left(m_{1},m_{2},l\right),$ is obtained by bilinear interpolation of the reference frame in the super-resolution grid.

### 2.3. Relationship between H and h

In this section, the relationship between H and h is deduced and validated, and the simulation experiment is designed to verify the correctness of the formula. The derived formula is proven to be suitable for SR reconstruction. In the process of SR reconstruction, the PSF of the HR image can be estimated by the PSF of the LR image; and $PSF_{high}=k\cdot PSF_{low}$, where the downsampling ratio is k.

When knife-edge areas are extracted from a remote sensing image with gray values from 0 to 1, the original signal along the gradient direction is represented by the unit step signal E. Given the PSF H and degrading and downsampling operator D, $\varepsilon^{\prime }$ is finally expressed as the signal in the image. Equation (8) can be determined in the first downsampling model:

(8) $$\varepsilon^{\prime }\left(x\right)=D_{k}\tilde{*}\left[E\left(x\right)*H\left(x\right)\right]$$

where $\tilde{*}$ is the downsampling operating symbol, the downsampling operator $D_{k}$ can be taken as a calculation of one-dimensional downsampling multiples of k, which is the equivalent of k compression from this function. The formula is shown in (9):

(9) $$D_{k}\tilde{*}f\left(x\right)=f\left(\frac{x}{k}\right)$$

Therefore, when the variable is less than 0, the signal value of E is 0; when the variable is greater than 0, the signal value is 1. With general downsampling using the convolution operation method, the knife-edge areas can be mathematically formulated as follows:

(10) $$\begin{array}{ll} \varepsilon^{\prime }\left(x\right) & =E\left(\frac{x}{k}\right)*H\left(\frac{x}{k}\right)\\ & =\;\int_{-\infty }^{+\infty }E\left(\frac{x}{k}-t\right)H\left(t\right)\;dt\\ & =\;\int_{-\infty }^{\frac{x}{k}}1\cdot H\left(t\right)\;dt+\int_{\frac{x}{k}}^{+\infty }0\cdot \;H\left(t\right)dt\;\\ & =\int_{-\infty }^{\frac{x}{k}}H\left(t\right)dt\\ & =\int_{-\infty }^{x}\frac{1}{k}H\left(\frac{t}{k}\right)dt \end{array}$$

According to the first downsampling model, Equation (11) can be deduced:

(11) $$\begin{array}{ll} g\left(x,y\right) & =D_{k}\tilde{*}\left[F\left(x,y\right)*H\left(x,y\right)\right]\\ & =D_{k}\tilde{*}\int_{-\infty }^{+\infty }\int_{-\infty }^{+\infty }F\left(x-u,y-v\right)H\left(u,v\right)du\;dv\\ & =\int_{-\infty }^{+\infty }\int_{-\infty }^{+\infty }\frac{1}{k^{2}}F\left(\frac{x-u}{k},\frac{y-v}{k}\right)H\left(\frac{u}{k},\frac{v}{k}\right)du\;dv \end{array}$$

Based on the principle of knife-edge method, the PSF can be obtained from the derivative function of the edge spread function (ESF). The PSF is calculated as shown in Equation (12) by the knife-edge method:

(12) $$\begin{array}{ll} h\left(x\right) & =\frac{d\varepsilon^{\prime }\left(x\right)}{dx}\\ & =\frac{1}{k}H\left(\frac{x}{k}\right) \end{array}$$

We can assume that the original signal can be restored effectively by the PSF with the values calculated by the knife-edge method. Deconvolution is used in the downsampling image g and the PSF initially. The deconvolution image requires rise sampling, so that the original image F can be obtained, as shown in Equation (13):

(13) $$F\left(x,y\right)=U_{\frac{1}{k}}\tilde{*}\left[g\left(x,y\right)⊗h\left(x,y\right)\right]$$

where ⊗ is the deconvolution operation. Upsampling operator $U_{\frac{1}{k}}$ can be taken as the calculation of one-dimensional upsampling multiples of $\frac{1}{k^{2}}$, which is the equivalent of the 1/k compression from the two-dimensional function of two coordinate axes, as shown in (14):

(14) $$U_{\frac{1}{k}}\tilde{*}f\left(x,y\right)=f\left(kx,ky\right)$$

If Equation (14) is correct, Equation (15) must exist:

(15) $$g\left(x,y\right)=\left[D_{k}\tilde{*}F\left(x,y\right)\right]*h\left(x,y\right)$$

Moreover:

(16) $$\begin{array}{ll} \left[D_{k}\tilde{*}F\left(x,y\right)\right]*h\left(x,y\right) & =F\left(\frac{x}{k},\frac{y}{k}\right)*h\left(x,y\right)\\ & =\int_{-\infty }^{+\infty }\int_{-\infty }^{+\infty }F\left(\frac{x-u}{k},\frac{y-v}{k}\right)h\left(u,v\right)du\;dv\\ & =\int_{-\infty }^{+\infty }\int_{-\infty }^{+\infty }\frac{1}{k^{2}}F\left(\frac{x-u}{k},\frac{y-v}{k}\right)H\left(\frac{u}{k},\frac{v}{k}\right)du\;dv \end{array}$$

Hypothesis (13) can be proven as tenable because Equations (11) and (16) yield the same results; hence, Equation (15) is correct.

The original signal can be restored effectively by the PSF of the existing degraded image proof. The signal can be applied in the process of image SR reconstruction. The PSF of the LR image calculated by the knife-edge method can be applied in the SR reconstruction process.

The PSF is approximated by Gaussian functions with appropriate parameters because the PSF follows a Gaussian distribution [27,28,29]. The PSF $H_{i}$ of the HR image can be written as follows:

(17) $$H_{i}\left(x\right)=\frac{1}{\sqrt{2\pi }\Sigma }e^{-\frac{1}{2\Sigma^{2}}x^{2}}$$

The PSF $h_{i}$ of the LR image can be expressed using Equation (18):

(18) $$h_{i}\left(x\right)=\frac{1}{\sqrt{2\pi }\sigma }e^{-\frac{1}{2\sigma^{2}}x^{2}}$$

Therefore, the relationship between the Gaussian function parameters and the PSF of the $H_{i}$ and $h_{i}$ images can be deduced from Equation (12), as shown in Equations (20), where Σ is the Gaussian function parameter of $H_{i}$, and σ is the Gaussian function parameter of $h_{i}$:

(19) $$\frac{1}{\sqrt{2\pi }\sigma }e^{-\frac{1}{2\sigma^{2}}x^{2}}=\frac{1}{k}\frac{1}{\sqrt{2\pi }\Sigma }e^{-\frac{1}{2\Sigma^{2}}{\left(\frac{x}{k}\right)}^{2}}$$

(20) σ=kΣ

**Figure 2:**
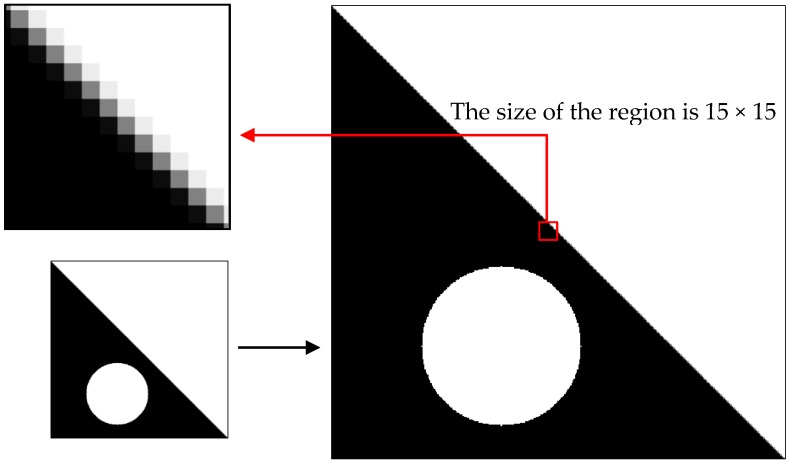
Selection process of knife-edge area. The resized image by resampling with multiples of k.

**Figure 3:**
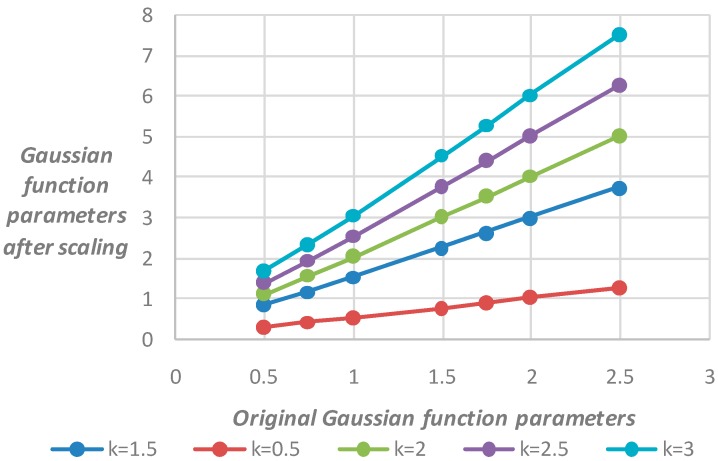
Relationship between the two parameters.

The simulation experiment is conducted to verify the proposed computation formula. A man-made knife-edge figure is drawn using computer language, and the figure is degraded by convolution by using the PSF, which is estimated by Gaussian functions. The selection process of the knife-edge area is shown in Figure 2. The seven Gaussian function parameters were set at 0.5, 0.75, 1.0, 1.5, 1.75, 2.0, and 2.5. The resized image by resampling is presented with different multiples of k set at 0.5, 1.5, 2.0, 2.5, and 3.0. The knife-edge area must be selected to obtain the Gaussian function parameter of the sampled image; the size of the region is 15 × 15 pixels. According to the formula derived, the result is in correspondence with the original parameter. The results are summarized in Table 1, and Figure 3 shows that the Gaussian function parameters between the original and scaled image basically meet the linear relationship.

The results in Figure 3 and Table 1 show the PSF Gaussian function parameters between the images before the downsampling and after satisfying the linear relationship when the image is scaled at different scales of k. The ratio of the scaling parameter variation to the original parameter variation is similar to k. The ratio satisfies the formula which was just deduced in Equation (20).

**Figure 4:**
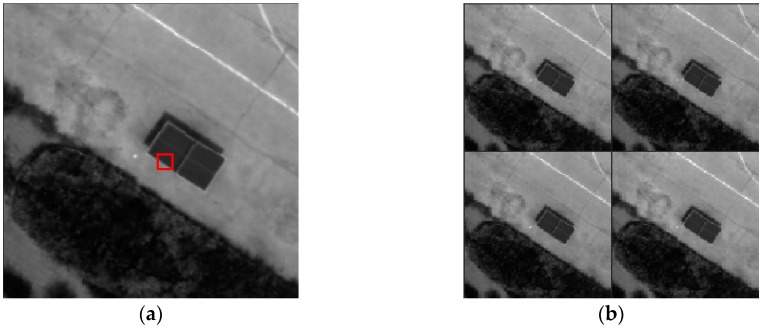

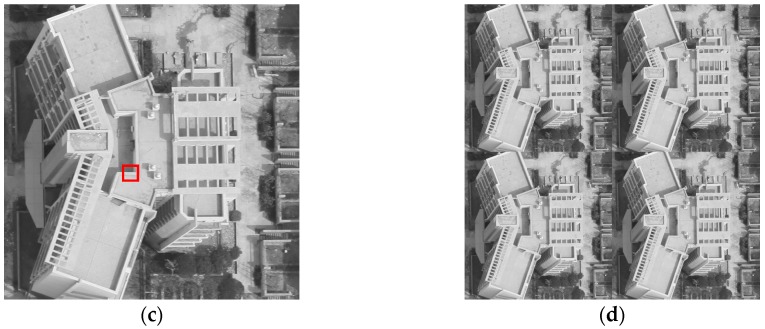
Experimental data. (**a**) Original ADS 40 image, the image enclosed in red box is the knife-edge area which is used to estimate the Gaussian function parameter (*σ*); (**b**) Four downsampled LR images of the ADS 40 image; (**c**) Original UAV image; (**d**) Four downsampled LR images of the UAV image.

The correctness of Equation (20) in the real image can be proven by the following experiments. The experimental images with some knife-edge areas can be selected by the ADS 40 remote sensing image with the size of 200 × 200 (Example 1) and the unmanned aerial vehicle (UAV) image with the size of 800 × 800 (Example 2). The experimental data are shown in Figure 4.

**Figure 5:**
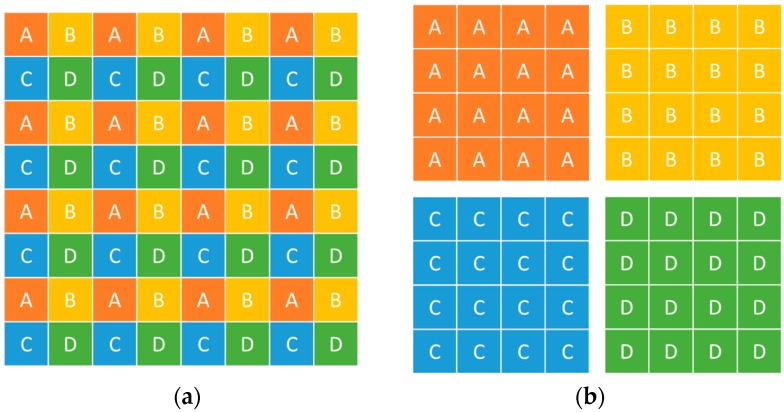
Graphic of a downsampling model. (**a**) Original image; (**b**) four downsampled LR images.

First, four LR images must be acquired from the downsampling model, as shown in Figure 5, in which the original image becomes a series of LR image sequences with size of 100 × 100.

Second, the knife-edge areas with four LR images and the experimental image are estimated using the PSF estimation based on slant knife-edge method. The PSFs can be obtained separately.

Finally, an oversampling rate is set at 2, indicating that the original image is zoomed out in half in the experiment. Based on the relationship between the images before the downsampling and after deducing, the Gaussian function parameter σ, after downsampling, must be half of the original. The results are shown in Table 2. The value coincides with the equation, proving that SR image reconstruction based on the PSFs of LR images is possible.

### 2.4. PSF Estimation of Low-Resolution Remote Sensing Images

The optical information of the remote sensing image is blurred in the process of the image capture because of the relative motion between the object being photographed and the satellite and the CCD or the atmosphere turbulence. The PSF of an imaging platform can represent the response of an imaging system to a point source. Therefore, the calculation of the PSF in the acquired image is a significant step to restore the ideal remote sensing images. The blurring process [30], as a convolution of an image, is shown in Equation (21):

(21) $$g\left(x,y\right)=f\left(x,y\right)*h\left(x,y\right)=\int_{-\infty }^{+\infty }\int_{-\infty }^{+\infty }f\left(\alpha ,\beta \right)\cdot h\left(x-\alpha ,y-\beta \right)d\alpha d\beta$$

where * is the convolution operator, h(x,y) represents the PSF, f(x,y) is the original image, g(x,y) is the degradation of the image, and α and β are the blurring filters.

**Figure 6:**
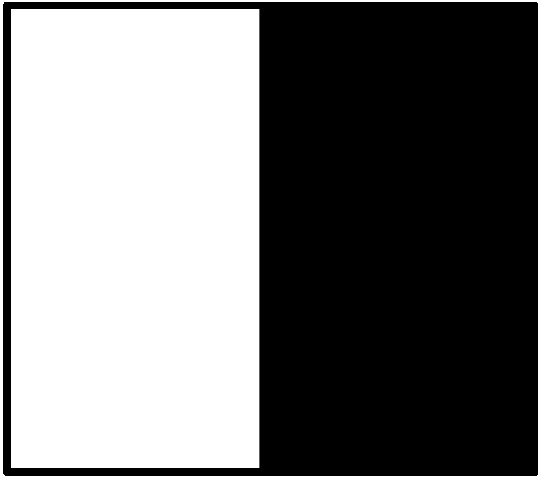
Ideal knife-edge area.

However, even when the PSF [31] is measurable, it is influenced by some unpredictable conditions; hence, many methods are proposed to solve the problem. The most common methods in PSF estimation are the knife-edge [32], spotlight [33], and pulse [34] methods. In addition, the knife-edge method is the most commonly employed method. The principles of the typical knife-edge method [35,36] and the ideal knife-edge area are shown in Figure 6. The knife-edge area is assumed as a square area, and the knife edge goes through the center of the area. Each row of the knife-edge satisfies formula (22), in which the value of the image greater than the edge boundary line $x_{0}$ is 1, and the value less than $x_{0}$ is zero [36,37]. However, the typical knife-edge method for point spread function estimation is limited by edge slant angle. The knife-edge in the image must be parallel to the sampling direction and the slant angle should be within 8° [32]. Under most circumstances, slant knife-edges have certain slope to the direction of the ideal ones; thus, the ideal knife-edge cannot be determined in all situations. Qin et al. [38] estimated the PSF by a robust method to solve this problem in the typical knife-edge method; they built a mathematical model of the relationship between the line spread function (LSF) estimated by the typical knife-edge method and the real PSF. Although an accurate PSF is obtained, the computed PSF still contains an error because the algorithm uses the discrete function to derive the subpixel error:

(22) $$f\left(x,y\right)=\{\begin{array}{c} 1\;x>x_{0}\\ 0\;x\leq x_{0} \end{array}$$

**Figure 7:**
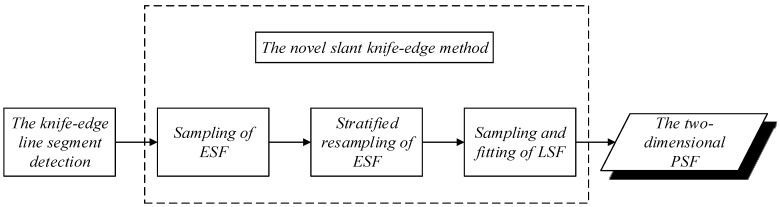
Process of the novel slant knife-edge method in a simplified sequence flow diagram.

In this study, a novel slant knife-edge method is used because this method fits the LSF directly and ensures the evenness of the edge spread function (ESF) sample to improve the accuracy of the PSF estimation. Figure 7 illustrates the process of the novel slant knife-edge method in a simplified sequence flow diagram. However, the area can be searched using several methods, and the three following requirements must be satisfied:

- The area cannot be extracted from the borders of the image to avoid the noise around the borders.
- The area must be excellent in linearity to ensure the accuracy of the PSF estimation.
- Evident gray value differences between two sides of the edge to reduce the influence of the noise must be obtained.

**Figure 8:**
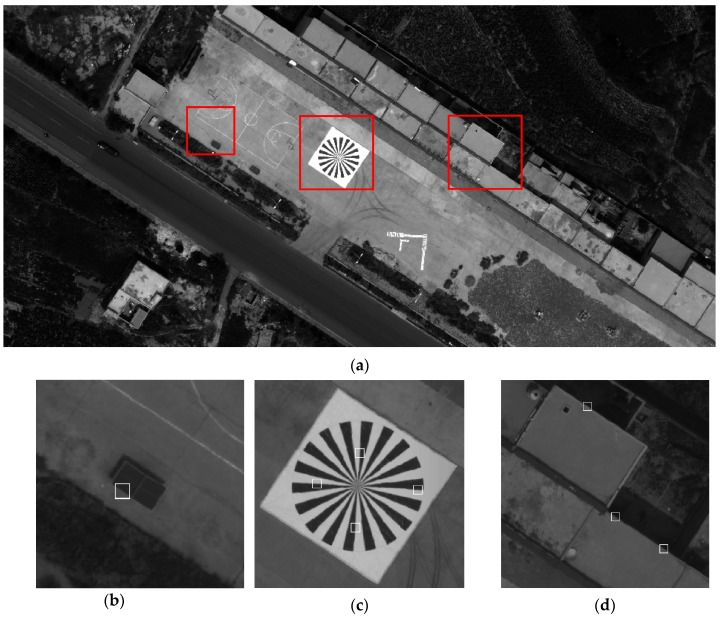
(**a**) Measurement area; (**b**); (**c**); and (**d**) are some examples of extracted knife-edge areas.

**Figure 9:**
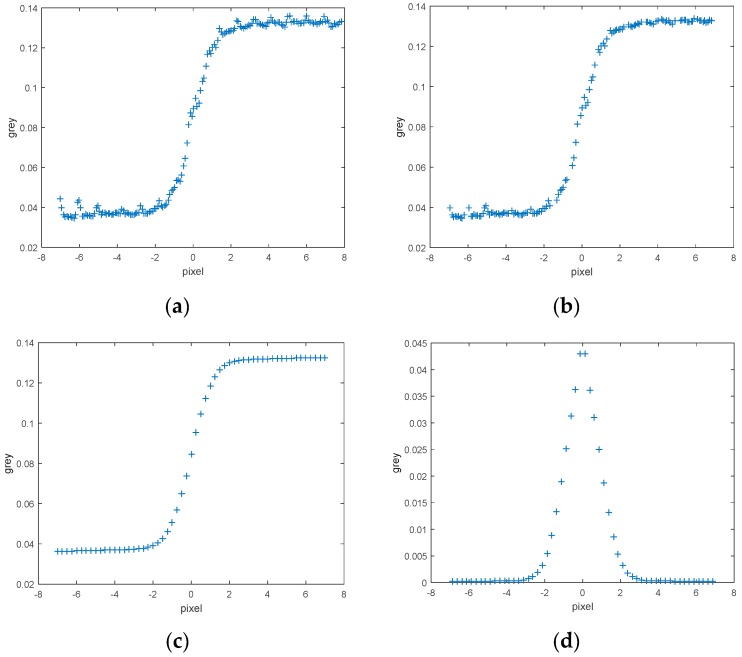
(**a**) Results of ESF sample; (**b**) ESF denoised sample; (**c**) ESF resample; (**d**) LSF sample.

The original data and results of the experiment are displayed in Figure 8. The measurement area and three examples of extracted knife-edge areas are also shown. Most of the knife-edge areas extracted successfully and the process of removing the weak-related areas are displayed. Follow ups are described in detail. ESF sampling, ESF denoising, ESF resampling, and LSF sampling results are shown in Figure 9.

## 3. Results and Discussion

### 3.1. Examples of Simulated Images

The experimental results did not demonstrate the effectiveness of the SR reconstruction algorithms qualitatively because of the lack of the ideal HR image. Therefore, the design of a simulation experiment is necessary by applying three different SR approaches and comparing the difference between these reconstructed images and the original image by the peak value signal-to-noise ratio (PSNR) and the mean square error (MSE) [39].

In this section, two series of comparative experiments are designed to evaluate the correctness of the deduced formula presenting the relation of the PSF Gaussian function parameter before and after SR reconstruction, as well as to compare the differences among the three reconstruction methods, namely, the proposed method, blind SR method, and bicubic interpolation method, on the original HR image. Moreover, given PSNR and MSE as the performance evaluation indicators, the experiment verifies the efficiency of the SR image reconstructed by the modified algorithm.

**Figure 10:**
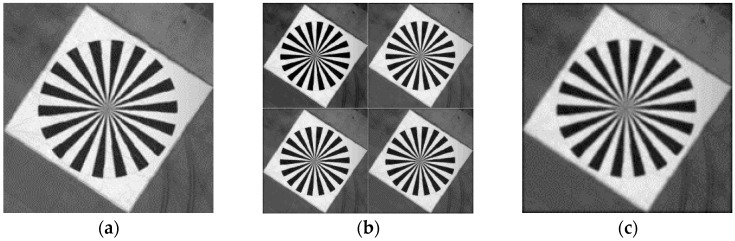
Simulation results. (**a**) Original image; (**b**) four simulated LR images; (**c**) one of the LR interpolated images, with the same size as that of the original image.

The experimental process is designed as follows:

1. The experimental image with some knife-edge areas can be selected by the ADS 40 remote sensing image with the size of 314 × 314, as shown in Figure 10a.
2. to the SR observation model, the original blurred image with given low pass and downsampled with factor 2 generated four LR images with the size of 157 × 157, as shown in Figure 10b. One of the LR simulated images is shown in Figure 10c. These four images correspond to the actual transformation parameters of the reference image, as shown in Table 3, where *Dx* represents the actual offset in the horizontal direction of the simulated LR image, and *Dy* is the actual offset in the vertical direction. *D*θ is the rotation angle because this experiment mainly considered translation; hence, the rotation angle is 0. The default units are pixels and degrees.
3. The knife-edge areas with four LR images are estimated using the PSF estimation based on slant knife-edge method, which is an accurate method. PSFs can then be obtained separately.
4. According to the derived formula (20), the PSF must be multiplied by the downsampling factor 2. Afterward, the POCS method with the estimated 2*PSF is used to reconstruct the HR image from these four downsampling LR images.
5. The blind SR and bicubic interpolation methods are used for the comparative experiments. The similarity between the experimental image and the resulting images of the three methods are observed.
6. Evaluation of experimental results.

**Figure 11:**
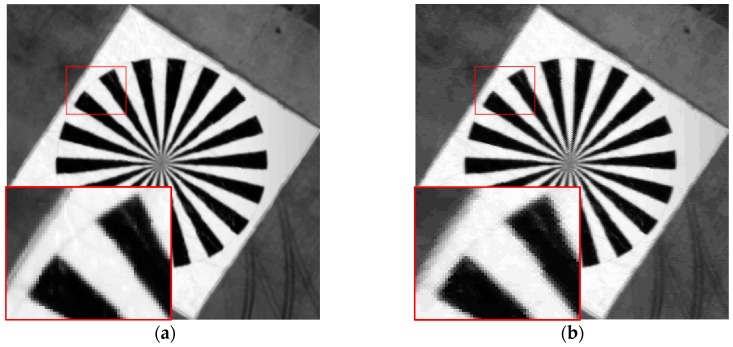

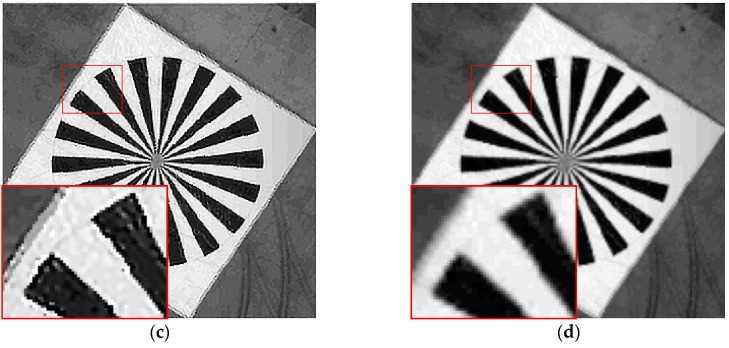
Simulation results of experiments with the three methods. The images enclosed in red box are the details of the three different algorithms. (**a**) Original image; (**b**) image based on the proposed algorithm; (**c**) image based on the blind SR reconstruction algorithm; and (**d**) image based on the bicubic interpolation algorithm.

No significant detailed difference between the original image and the modified reconstructed image in Figure 11 can be observed. The results testify that the algorithm can effectively reconstruct images. However, the details of the resulting images of the blind SR reconstruction algorithm and the bicubic interpolation algorithm are inconsistent with the original image in terms of heavy noise, aliasing, excessive sharpening, and so on. Nevertheless, both algorithms can sharpen edges and receive large amounts of information.

Table 4 demonstrates that the proposed method contains less signal distortion and substantially surpasses the blind SR reconstruction and the bicubic interpolation algorithms in terms of the highest PSNR and the minimum MSE. Moreover, the results prove that the POCS method with the estimated PSF can improve the recovery image information and achieve a good performance of SR reconstruction.

### 3.2. Examples of Real Images

In this section, the proposed algorithm is applied in practice. The experiments are designed to investigate the effect of SR reconstruction of the POCS method with the estimated PSF in the agricultural application. Given that data sources in agriculture use HR remote sensing images, the agricultural region of the GF-2 image is selected as the experimental data with the image size of 400 × 400. The set of LR inputs are the overlapped areas of two adjacent images. The other data are the UAV images with size of 500 × 500 to prove the stability of the proposed algorithm. The experimental results are compared with the blind SR reconstruction and the bicubic interpolation reconstruction to verify the effectiveness. Because the reference image is unavailable, the quality metrics PSNR or MSE cannot be used to compare the advantages of the three algorithms. Therefore, we choose a no-reference metric Q, which can react in a natural way to the presence of noise and blur, to provide a quantitative measure of true image. And its value drops means the variance of noise rises, and the image becomes blurry [40]. Simultaneously, manual visual interpretation also is a criterion method.

**Figure 12:**
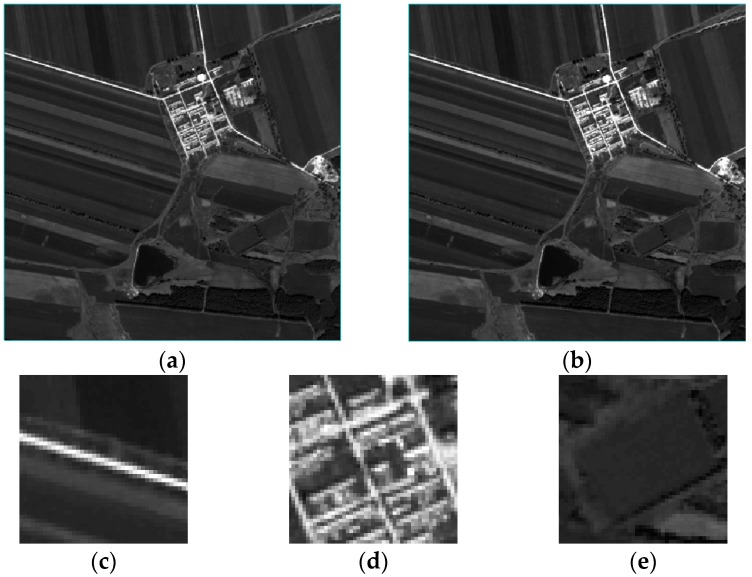
(**a**,**b**) are the experimental data; (**c**) detail of the road; (**d**) detail of the house; (**e**) detail of the paddy fields.

In the first set of experiments, the knife-edge areas are extracted using the novel slant knife-edge method. The experimental data and details are shown in Figure 12. However, the edge of the agriculture land is unclear, and the house is fuzzy, thereby preventing the researchers from surveying the area accurately.

**Figure 13:**
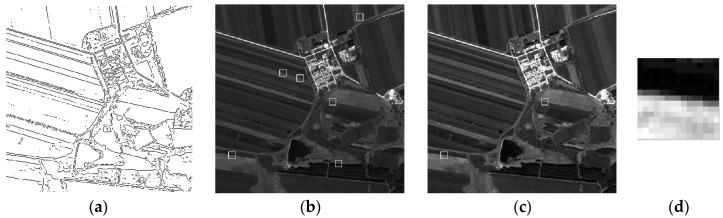
Process of extracting the edge region by using the novel slant knife-edge method. (**a**) Edge detection by canny algorithm; (**b**) all knife-edge areas are described in white boxes; (**c**) preferred knife-edge areas; (**d**) selected knife-edge area.

The knife-edge areas are shown in Figure 13. The knife-edge region selection is based on the relevant center coordinate of the knife-edge area. Two knife-edge areas are selected (c) from a total of six knife-edge areas (b), as shown in Table 5. Experimental data show that the center coordinates [36, 320] of the region is the most clear knife-edge area. Therefore, with the point as the center, a square is drawn with a radius of 7 pixels to select the area, which is shown in (d). Given these data, the PSF is calculated for subsequent reconstruction experiments.

**Figure 14:**
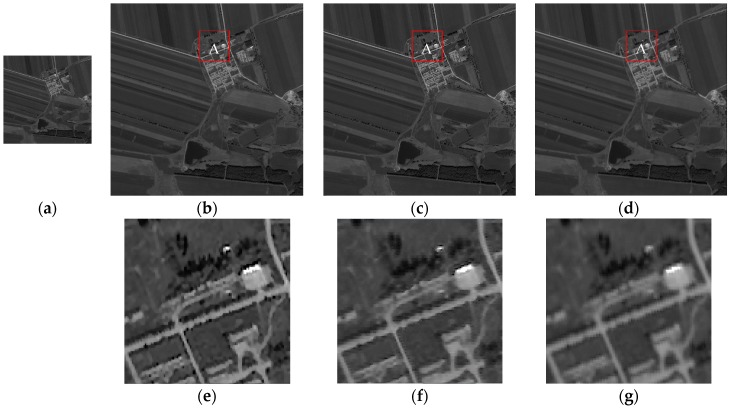
Actual results of the experiments using the three methods. The images enclosed in a red box are the details of the three different algorithms. (**a**) Original LR image; (**b**) image based on the proposed algorithm; (**c**) image based on the blind SR reconstruction algorithm; (**d**) image based on the bicubic interpolation algorithm; (**e**) is the detail of reconstruction in region A by the proposed algorithm; (**f**) is the detail of reconstruction in region A by the blind SR reconstruction algorithm; and (**g**) is the detail of reconstruction in region A by the bicubic interpolation algorithm.

Subsequently, the two selected PSFs are taken into the POCS algorithm to obtain the reconstructed image. The nominal values of metric Q of three reconstructed images as shown in Table 6. Metric Q of the image based on the proposed algorithm are the maximum number, it shows that the algorithm has a good visual performance and detail preservation. What’s more, Figure 14 shows the results and details of the HR image reconstruction. In region A, the edges of the building are clear and distinct in (e) and (f). The bicubic interpolation method can only interpolate, but the interpolation is unclear. Moreover, the images reconstructed by the blind SR reconstruction method present jagged edges. Therefore, the proposed method and the blind SR reconstruction method can effectively reconstruct the details of the building. In summary, the results indicate that all these algorithms can effectively produce robust SR images. However, the proposed method demonstrated better effect than the other algorithms.

**Figure 15:**
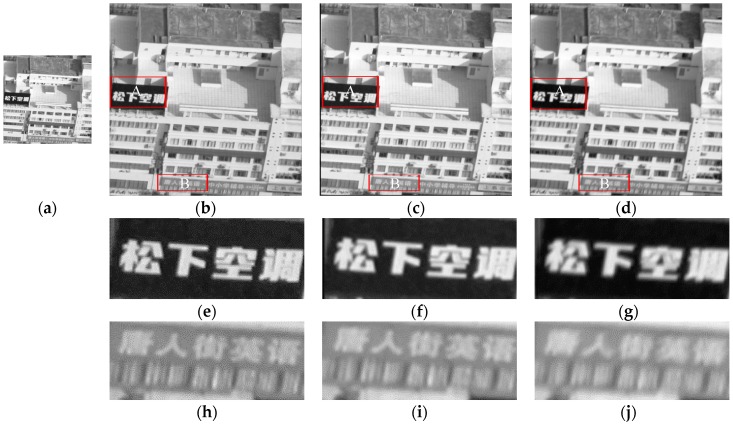
Actual results of the second set of experiments using the three methods. The images enclosed in a red box are the details of the three different algorithms. (**a**) Original LR image; (**b**) image based on the proposed algorithm; (**c**) image based on the blind SR reconstruction algorithm; (**d**) image based on the bicubic interpolation algorithm; (**e**) is the detail of reconstruction in region A by the proposed algorithm; (**f**) is the detail of reconstruction in region A by the blind SR reconstruction algorithm; (**g**) is the detail of reconstruction in region A by the bicubic interpolation algorithm; (**h**) is the detail of reconstruction in region B by the proposed algorithm; (**i**) is the detail of reconstruction in region B by the blind SR reconstruction algorithm; and (**j**) is the detail of reconstruction in region B by the bicubic interpolation algorithm.

In the second set of experiments, we choose the most clear knife-edge area [114, 329] in real images according to the previous experimental procedure. The UAV images, as well as the results and details of the HR image reconstruction, are shown in Figure 15 and the values of metric Q are shown in Table 7. The reconstruction results of the proposed method are the most natural; the image becomes clearer with the increase in the amount of image information. Although the blind SR algorithm can achieve a certain reconstruction effect, the edge of the reconstructed image is not sharper than the image obtained using the proposed method.

## 4. Conclusions

In summary, the POCS method with the estimated PSF based on multiple LR images describes a number of key initiatives including the improvement of the accuracy of the PSF of LR images by a novel slant knife-edge method. The validity and reliability of the formula, which derives the relationship between the images before and after downsampling, have been proven. The value of the downsampling multiplied by the PSF of LR images is equal to the estimated PSF of the HR image. The formula can be applied in image restoration and SR reconstruction. The formula can also enhance the clarity in agricultural remote sensing images. Finally, the estimated PSF was combined with the POCS method to improve the accuracy of the SR reconstruction process. Our experimental results show that the deduced formula of PSF is accurate and significant in the development of the restoration and reconstruction processes. However, some problems remain to be solved. The quality of the knife-edge area significantly influences the estimation accuracy of the PSF, leading to some errors.

## Figures and Tables

**Table 1:** Gaussian function parameters before and after scaling.

		*k*	*k* = 0.5	*k* = 1.5	*k* = 2	*k* = 2.5	*k* = 3
	*σ*						
∑							
0.5			0.3154	0.8556	1.1164	1.401	1.6878
0.75			0.4375	1.1739	1.5565	1.944	2.3341
1			0.5476	1.5337	2.0346	2.5461	3.0514
1.5			0.7861	2.2715	3.0206	3.7757	4.5295
1.75			0.9101	2.642	3.5195	4.3967	5.2731
2			1.0336	3.0144	4.0175	5.019	6.0212
2.5			1.2775	3.7623	5.0135	6.2669	7.5232

**Table 2:** Gaussian function parameter (σ) of the image before and after downsampling.

	Original Image (*σ*)	Estimate of Downsampling Image (*σ*/2)	Real Result of Each Downsampling Image
Example 1	0.5894	0.2992	0.2979
			0.3067
			0.3102
			0.3098
Example 2	0.3809	0.1905	0.2235
			0.2158
			0.1884
			0.1923

**Table 3:** Transformation parameters of the simulation image.

	*Dx*	*Dy*	*Dθ*
1	0.5	0.5	0
2	1.2	1.2	0
3	0.4	0.4	0
4	1.8	1.8	0

**Table 4:** Evaluation results of the experiment.

Image	PSNR	MSE
Image based on the proposed algorithm	96.7904	$1.3616\times 10^{-5}$
Image based on the blind SR reconstruction algorithm	54.5366	$2.2884\times 10^{-1}$
Image based on the bicubic interpolation algorithm	81.8529	$4.2441\times 10^{-4}$

**Table 5:** List of the center coordinates of knife-edge areas.

Number	[X, Y]	Relevant
1	[36, 320]	0.9825
2	[249, 207]	0.9763
3	[144, 146]	0.9748
4	[180, 157]	0.9722
5	[261, 338]	0.9557
6	[306, 28]	0.9187

**Table 6:** The nominal values of metric Q of three reconstructed images in the first set of experiments.

Image Reconstructed by Different Algorithms	The Value of *Q*
the proposed algorithm	24.2285
the blind SR reconstruction algorithm	21.6771
the bicubic interpolation algorithm	17.3591

**Table 7:** The nominal values of metric Q of three reconstructed images in the second set of experiments.

Image Reconstructed by Different Algorithms	The Value of *Q*
the proposed algorithm	40.1082
the blind SR reconstruction algorithm	34.8491
the bicubic interpolation algorithm	28.3006
